# Success and efficiency of phase 2/3 adjunctive trials for MDD funded by industry: a systematic review

**DOI:** 10.1038/s41380-020-0646-3

**Published:** 2020-01-27

**Authors:** Naji C. Salloum, Maurizio Fava, Sophia Ball, George I. Papakostas

**Affiliations:** Clinical Trials Network and Institute, Massachusetts General Hospital, Boston, MA USA

**Keywords:** Depression, Neuroscience

## Abstract

To review the success rate and efficiency of industry-sponsored phase 2/3 clinical trials for adjunctive therapies for antidepressant partial- and non-responders with major depressive disorder (MDD), a systematic search of Pubmed/Medline was conducted, in addition to abstracts of major psychiatric meeting held since 2010, of randomized, placebo-controlled adjunct oral pharmacotherapy trials in this patient population. Forty-six (*n* = 33,900; 70 drug compactor arms) trials were pooled, yielding only three approved drugs. Twenty-two (31.4%) drug-placebo comparisons were successful. Numerically, success rates for treatment arms from studies with one versus more than one drug-placebo comparison were higher (39.3% versus 26.2%). The antidepressant lead-in employing single-blind placebo and the sequential-parallel comparison design (SPCD) were successful in 50% and 40% of cases, respectively. The direct randomization (no lead-in) design yielded positive results in one third of cases. The success rate of open-label antidepressant lead-ins without placebo or using double-blind placebo was very poor (<15%). There was also a pronounced discrepancy in terms of efficiency across study designs. Accounting for sample size requirements, a phase 3 program using SPCD would have a higher cumulative chance of yielding two positive trials (50%) than a phase 3 program using a single-blind placebo lead-in (40%). Future programs should carefully weigh the need for a lead-in, which is time-consuming, expensive and, in some cases (i.e., open-label antidepressant without placebo or with double-blind placebo) nearly futile. Instead, more effort should involve the use of studies where patients are directly randomized, such as the SPCD, with more investment shifted towards the accurate and independent vetting of subject eligibility.

## Introduction

Major depressive disorder (MDD) is a highly prevalent and debilitating chronic illness, associated with functional impairment and poor quality of life [1]. MDD is a treatable disease, with a wide number of compounds approved on the market [2]. However, almost all compounds are approved as monotherapy, and it has been well established that only a subset of patients remit following one or more trials with these agents [3]. Thus, to succeed, clinicians often resort to adjunctive treatment [4]. However, only three compounds have, thus far, been approved as adjunct therapy in the U.S. (quetiapine, aripiprazole, and brexpiprazole), and one in the E.U. (quetiapine). To make matters worse, all three agents belong to the same pharmacological class of atypical antipsychotics, known to carry a significant side-effect burden [5–7]. While two additional therapies, the olanzapine plus fluoxetine combination and ketamine [8, 9], exist for the indication of treatment-resistant depression (TRD) (defined by the U.S. Food and Drug Administration MDD with failure to respond to two or more antidepressant trials [10]), these are not approved as adjunctive therapy nor studied as second-line treatments. Given the popularity of adjunctive pharmacotherapies in MDD (including second-line) [4], there is a clear need to further develop effective, safe, and well-tolerated adjunctive medications for this indication.

The process necessary to generate an approved treatment for MDD in any indication (monotherapy, adjunct, TRD) is an expensive and arduous endeavor, involving massively expensive clinical trials, and often resulting in unsuccessful or prematurely terminated programs unable to consistently demonstrate the efficacy of the investigational compound. Two main reasons exist for this: the compound is truly not effective in treating the illness (negative study), or the clinical trial was designed in such a way that could not demonstrate whether the treatment effect truly exists (failed or uninformative study) [11]. Multiple factors have been identified as possible contributors to uninformative studies including elements of study design, inclusion/exclusion criteria, an unpredictable and sizeable placebo response rate, the inclusion of inappropriate study subjects, or the choice of primary outcome measure [12, 13]. When designing trials for adjunctive treatments in MDD, there is the additional complexity of defining the population as being partial- or non-responders to pharmacotherapy and assessing the degree of historical treatment resistance, as this is a critically important methodological aspect [14]. This can be done either historically (retrospectively, as in the quetiapine phase 3 adjunctive program) or with the use of lead-in (prospectively, as in the cases of aripiprazole and brexpiprazole). A third option includes the use of the sequential-parallel comparison design (SPCD), which is a hybrid of the first two methods [15]. Until recently, however, there has been very little systematic research in this area. Therefore, our goal was to review phase 2/3 clinical trials of adjunctive oral pharmacotherapies for MDD funded by industry with a focus on comparing their success, efficiency, and placebo response rates as a function of lead-in use and type.

## Methods

### Data sources and search strategy

Phase 2/3 clinical trials of adjunctive oral pharmacotherapies for MDD funded by industry were first identified using searches of Pubmed/Medline. An initial search was conducted by cross-referencing the terms “depressive”, “randomized”, and “adjunctive”. A follow-up search was then conducted by cross-referencing the terms “depressive”, “randomized”, and “augmentation”. Finally, a third search was conducted by cross-referencing the terms “depressive”, “randomized”, and “add-on”. These searches were limited to studies published in the last 15 years (Until March 2019). We also obtained the program syllabi and searched the abstracts of major psychiatric meetings held since 2010 (American Psychiatric Association; American Society of Clinical Psychopharmacology; European College of Neuropsychopharmacology; Collegium Internationale Neuropsychopharmacologicum; Society of Biological Psychiatry, World Federation of Societies of Biological Psychiatry; World Psychiatric Association; International Society for Affective Disorders). Authors or study sponsors were contacted to obtain a copy of the presentation, as well as any pertinent study details for unpublished studies. For multiple poster presentations of an unpublished trial, the most recent presentation was used.

### Study selection

We selected for randomized, double-blind, placebo-controlled studies evaluating the acute efficacy of adjunctive treatments to antidepressants for adults MDD who are antidepressant partial- or non-responders.

We then selected studies that also met the following inclusion criteria:

1. Used either the Hamilton Depression Rating Scale (HDRS [16]) or the Montgomery–Asberg Depression Rating Scale (MADRS [17]) as their primary outcome measure.
2. Exclusively focused on patients with MDD.

Studies where the augmented antidepressant was either not stable for a period of at least 4 weeks before randomization or during the double-blind period were excluded, as were studies focusing on drugs not in their oral formulation, studies focusing on devices, or studies not primarily funded by the Pharmaceutical Industry. Studies with a double-blind treatment duration <4 weeks were also excluded, as were studies where the primary outcome was earlier than one-week post-randomization.

### Data extraction

Data were extracted with the use of a precoded form. The following data were extracted from studies included in the analysis: the number of patients randomized to each treatment arm and number of treatment arms, the design of the trial with respect to the use of antidepressant lead-ins (i.e., none, open-label, single-blind placebo, double-blind placebo, sequential parallel comparison design, or SPCD), the duration of the lead-in and of the randomized portion of the trial, medication and dosing, the primary outcome measure used (HDRS or MADRS), trial arm success on primary outcome measure, and mean change in MADRS or HDRS scores from baseline.

### Quantitative data synthesis

The main outcome of this analysis was to estimate the proportion of drug treatment arms which met their a-priori-defined primary outcome both overall, as well as by design characteristics. A secondary aim was to examine the prevalence of various design practices across the entire adjunctive trial program funded by Industry, and to calculate the efficiency of each design. Efficiency was defined as the number of subjects enrolled in a study, either to participate in a lead-in or to be directly randomized, divided by the number of drug arms for that study.

## Results

Initially, 1066 results identified by the three successive Pubmed/Medline searches were reviewed. Of these, 989 involved trials not exclusively focusing on antidepressant partial- and non-responders, focused on different medical conditions or healthy volunteers, were reviews, opinions, surveys, chart reviews, case studies/series, uncontrolled biomarker studies that did not meet the inclusion criteria, or were simply duplicate or triplicate results identified by more than one search combination. The remaining 77 distinct abstracts described double-blind, randomized experiments in antidepressant partial- and non-responders with MDD utilizing either the HAMD or MADRS as the primary outcome measure. Fourteen of these manuscripts were excluded because they described studies where the augmented antidepressant was either not stable for a period of at least 4 weeks before randomization or during the double-blind period. Nineteen manuscripts were excluded because they either described studies focusing on drugs not in their oral formulation, or because they were focusing on devices. One was excluded because it described a study with a double-blind treatment duration of less than 4 weeks. Finally, nine manuscripts were excluded because they were not primarily funded by a pharmaceutical sponsor. The remaining 34 manuscripts described clinical trials eligible for inclusion. Three additional unpublished eligible trials presented at major international meetings were also identified, one of which was updated by the sponsor to be in press at the time of this search. The full list of included trials and their characteristics is available in supplemental Table 1. Using a modified rating scheme from the Oxford Center for Evidence-based Medicine for ratings of individual studies, the quality of the evidence in all included studies was one, referring to “properly powered and conducted randomized clinical trial”.

**Fig. 1:**
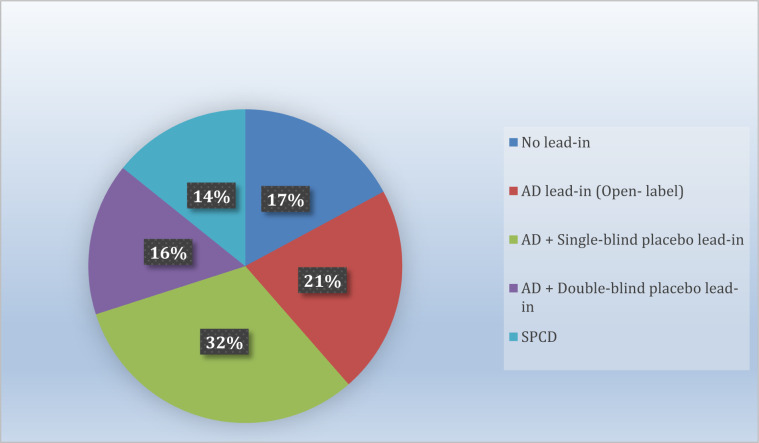
Study arm distribution by design. AD antidepressant; SPCD sequential parallel comparison design.

We were able to obtain data on whether individual treatment arms met the a-prior-defined primary outcome for all treatment arms across all studies. Thus, the systematic review was all-inclusive. Overall, a total of 46 double-blind, randomized clinical trials comparing adjunctive pharmacotherapies versus adjunctive placebo in patients with MDD who were partial- or non-responders to antidepressants were pooled (see supplemental Fig. 1). These trials contained a total of 33,900 subjects enrolled in a total of 70 pharmacotherapy-placebo pairwise-comparisons. Twelve of these comparisons did not involve the use of a lead-in, but exclusively relied on a retrospective assessment of antidepressant partial/non-response status and pre-randomization antidepressant dose stability. The remainder employed the following type of lead-in: either an antidepressant alone (open-label) (*n* = 15), or an antidepressant plus either single-blind (*n* = 22), or double-blind (*n* = 11) placebo. Finally, ten employed the SPCD design (Fig. 1) [15]. Supplemental Fig. 2 illustrates the different designs of studies included in this report. Characteristics of pooled drug treatment arms are listed in Table 1.

**Table 1:** Characteristics of Pooled Trials.

	Mean (sd), [Range]
Year Published	2015 (3.1), [2007–2019]
Duration of lead-in (weeks)	6.7 (2.2), [2–10]
Duration of randomized portion (weeks)	6.7 (2.4), [4–24]
	***N*** **(%)**
MADRS as primary outcome	42 (91)
Atypical Antipsychotics	21 (46)
Other Monoaminergic Agents	9 (20)
Opioid Modulators	4 (9)
Glutamate Modulators	2 (4)
Acetylcholine Modulators	5 (11)
Other Mechanism of Action	5 (11)

*MADRS* Montgomery–Asberg Depression Rating Scale (Montgomery and Asberg, 1979).

**Fig. 2:**
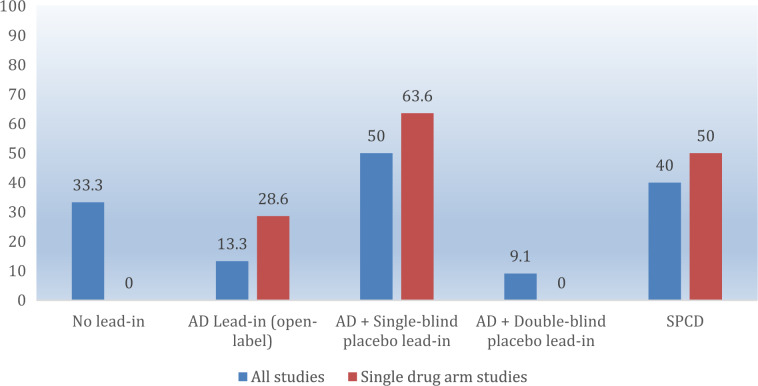
Percent study arm success across designs. AD antidepressant, SPCD sequential parallel comparison design.

**Fig. 3:**
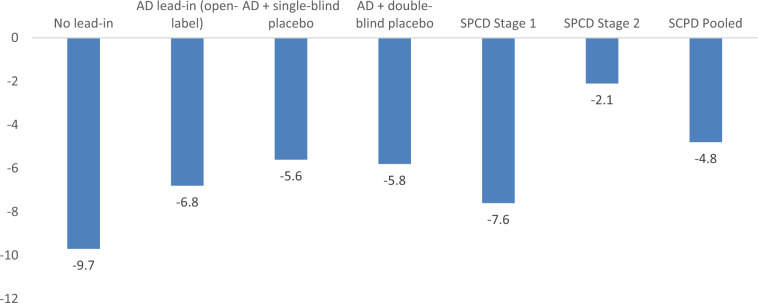
Reduction in depression severity scores MADRS or HAMD17) on placebo at week 4. AD antidepressant, SPCD sequential parallel comparison design.

**Fig. 4:**
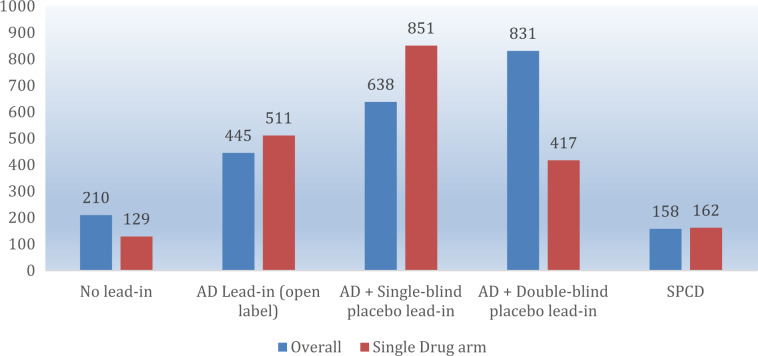
Average number of subjects enrolled per drug arm across study designs. AD antidepressant, SPCD sequential parallel comparison design.

Twenty-two of 70 (31.4%) comparisons achieved statistical significance on the study pre-defined primary outcome. Success rates across the five designs are shown in Table  2. Success rates focusing on studies with only one drug-placebo comparator across the five designs are shown in Fig. 2, while mean symptoms (MADRS or HDRS) reduction on adjunctive placebo during the randomized phase is shown in Fig. 3 (note: there was only a single trial that employed a 1:1, drug:placebo design without the use of a lead-in). Finally, number of enrolled and treated patients per comparator arm across the study designs is depicted in Fig. 4. Numerically, success rates for treatment arms from studies with one versus more than one drug-placebo comparison were higher (11/28 = 39.3% versus 11/42 = 26.2%). The antidepressant lead-in employing single-blind placebo was successful in 50% of cases (11/22). The SPCD also proved quite successful 40% of cases (4/10), while the direct randomization (no lead-in) design yielded positive results in one third of cases (4/12). The track record for open-label antidepressant lead-ins without placebo or using double-blind placebo was very poor (2/15 = 13.3%, 1/11 = 9.1%, respectively). There was also a pronounced discrepancy in terms of efficiency across study designs. Accounting for sample size requirements, a phase 3 program using SPCD with one drug comparator would have a higher cumulative chance of yielding two positive trials (50%) than a phase 3 program using a single-blind placebo lead-in with one drug comparator (40%), as detailed further in the discussion section.

## Discussion

The present work represents the most updated and comprehensive effort conducted thus far, attempting to understand how success and efficiency may vary across the body of industry-sponsored phase II and III clinical trials aimed at developing oral adjunctive drugs for MDD partial- and non-responders to standard therapy. Perhaps the most staggering finding is the sheer size of this effort. Specifically, in little over 12 years, nearly 34,000 subjects have been enrolled and treated in adjunctive drug trials, either directly randomized to add-on investigational drug versus placebo, or enrolled in a lead-in prior to randomization. Unfortunately, however, this effort has led to the approval of only three (in the case of the U.S.) or one (in the case of the E.U.) medications with this indication. In contrast, an older meta-analysis focusing on monotherapy trials in MDD which were conducted from 1980 to 2007 involved over 35,000 subjects randomized to either placebo or more than a dozen approved drugs [18].

Although the overall chances of “success” of a drug treatment arm, defined as meeting the a priori-defined primary outcome versus placebo, was estimated as, approximately, one-in-three, interesting patters emerged when studies are subtyped according to certain design elements. We propose that these patterns serve as lessons going forward. First, it is noted that studies which employ only a single drug-placebo comparator are more successful than those that employ more. This extends our previous findings from monotherapy studies [18] where more active treatment arms correlated with smaller drug-placebo effect sizes, likely due to an enhanced “placebo” effect in the latter group stemming from participant’s awareness than the chances of receiving placebo are lower [13]. Hence, future studies should, in the overwhelming majority of cases, focus on a single active drug arm per study. Second, not all “lead-in” designs appear to be equally successful. While the antidepressant lead-in employing single-blind placebo was successful in 50% of cases, the track record for open-label antidepressant lead-ins without placebo or using double-blind placebo lead-ins was very poor. Hence, these latter two trial designs should be avoided completely. Hence, while an earlier and more limited (*n* = 4,676) review had suggested that adjunctive studies without a lead-in could perform as well as those that did employ an antidepressant lead-in [19], accumulating data are beginning to show better performance for the antidepressant plus single-blind lead-in design. The SPCD design also proved quite successful (40% rate of positive treatment arms), while the direct randomization (no lead-in) design yielded positive results in only one third of cases. Finally, mean symptom score (MADRS or HDRS) reductions during the randomized portion of trials corroborates what is seen in Table 2, with the two most successful designs (antidepressant lead-in employing single-blind and SPCD) having the lowest placebo response rates—a pattern common among MDD trials [12].

**Table 2:** Study Designs Success Rates.

	Total number of arms	Total number of individual trials	Number of arms that met primary endpoint	% of arms that met primary endpoint	Total N enrolled	Placebo primary outcome (MADRS or HAMD) mean change
All Studies	70	46	22	31.4	33990	−7.6
SPCD	10	7	4	40.0	1585	−5.1
Non-SPCD	60	39	18	30.0	32405	−8.4
No Lead-in non-SPCD	12	6	4	33.3	2530	−12.2
OL lead-in non-SPCD	15	10	2	13.3	6688	−10
SB lead-in non-SPCD	22	15	11	50.0	14043	−6.3
DB lead-in non-SPCD	11	8	1	9.1	9144	−7.7
One drug arm	28	28	11	39.3	15816	−7.4
Two or more drug arms	42	18	11	26.2	18174	−9.1
One drug arm SPCD	4	4	2	50.0	651	−4.4
One drug arm no lead-in non-SPCD	1	1	0	0.0	129	−10.3
One drug arm OL lead-in non-SPCD	7	7	2	28.6	3580	−9.7
One drug arm SB lead-in non-SPCD	11	11	7	63.6	9368	−6.4
One drug arm DB lead-in non-SPCD	5	5	0	0.0	2088	−7.9

*SPCD* sequential-parallel comparison design, *OL* open-label, *SB* single-blind, *DB* double-blind, *MADRS* Montgomery–Asberg Depression Rating Scale, *HAMD* Hamilton Depression Rating Scale.

Choosing the right study design for a phase III program, however, is not simply an intellectual exercise, but requires a practical and pragmatic approach. Here, patterns of differential efficiency emerge across trials based on design characteristics. Specifically, average sample sizes per drug arm are severalfold higher for studies with versus without a lead-in. And since drug approval is dependent on the replication of success, estimating sample sizes and chances of success of a phase III program as a function of efficiency can be highly informative. For instance, in the hypothetical example where one sponsor choses to conduct two phase III studies utilizing the single-blind placebo lead-in with only one drug comparator, the estimated total sample size would be, approximately, 1700 subjects (a cumulative sample size of 9368 subjects was used in the 11 one-arm single-blind lead-in trials included in this report) and the program would have a nearly 40% chance of success (0.63*0.63; because of 63% success rate for each trial using a one-arm single-blind placebo lead-in design). In contrast, a program involving three SPCD studies with a single drug comparator would require just over 25% (*n* = 488; a cumulative sample size of 651 subjects was used in the 4 one-arm SPCD trials included in this report) of that sample, yielding a cumulative chance of program success (defined as at least two of three phase III trials being positive) of 50% (because of 50% success rate for each trial using a one-arm SPCD design). Feasibility of enrollment and time to study start and completion aside, cost savings in the latter scenario (smaller sample size) would be further compounded by trial design differences (lead-in studies are longer in duration and, hence, more expensive). Perhaps most importantly, from a human subjects’ perspective, there is also the need to consider the smallest possible number of patients that would preserve adequate statistical power when designing or approving a study. Going forward, all these factors should be taken into context when contemplating a pivotal trial program.

There are several limitations to our work. First, our focus was on the use of oral agents as adjunctive therapies for MDD. It cannot be assumed that our findings would extend to other indications (i.e., TRD), routes of administration where placebo responses may differ (i.e., intranasal, intravenous), or different modalities (i.e., monotherapies). Second, the range of medications studied across trials was relatively broad, with only a small subset of those approved. This rules out using the degree of drug-placebo “separation” in each trial as a valid measure of comparison across trials. In the future, as data accumulates, a formal meta-analysis should be conducted examining the impact of study design on the subset of studies involving FDA-approved agents only and, ideally, in similar populations and employing similar doses. Third, our findings extend to industry-sponsored studies but not necessarily to studies funded by other sources. In our review, we exclusively focused on industry-sponsored trials because they are more similar to each other than nonindustry studies, which are typically underpowered, employ less rigorous rater training and surveillance, and do not typically employ independent subject vetting for inclusion/exclusion. Finally, our efforts to identify missing trials were extensive. However, it is quite possible that we may have accidentally omitted to include a few trials.

In conclusion, despite great efforts from industry, very few oral drugs have thus far been approved as adjunctive therapy in antidepressant partial- and non-responders with MDD. Existing studies vary in terms of success and efficiency, serving as a valuable opportunity to learn from past mistakes. The past five years have witnessed an unprecedented growth in diversification of possible therapeutic targets and capital investment in the adjunctive MDD investigation venture [20, 21]. However, at the same time, several of program failures have prompted a number of Industry sponsors to announce their exit from the area of MDD or the field of Psychiatry altogether, making it painfully clear that stakeholders involved in treatment development in this therapeutic area urgently need to critically revamp the design and conduct of adjunctive clinical trials in order to maintain the necessary momentum for success and prevent a sense of futility from setting in. Future programs should carefully weigh the need for a lead-in, which is time-consuming, expensive and, in some cases (i.e., open-label antidepressant without placebo, or with double-blind placebo lead-in) futile. Instead, more effort should involve the use of studies where patients are directly randomized such as the SPCD, with more investment shifted towards the accurate and independent vetting of subject eligibility to compensate for the removal of the lead-in observation period.

## Supplementary information

Supplemental Table 1

Supplemental Figure 1

Supplemental Figure 2

## References

[1] Kessler RC, Berglund P, Demler O, Jin R, Koretz D, Merikangas KR (2003). The epidemiology of major depressive disorder: results from the National Comorbidity Survey Replication (NCS-R). JAMA..

[2] Papakostas GI (2010). The efficacy, tolerability, and safety of contemporary antidepressants. J Clin Psychiatry.

[3] Rush AJ, Trivedi MH, Wisniewski SR, Nierenberg AA, Stewart JW, Warden D (2006). Acute and longer-term outcomes in depressed outpatients requiring one or several treatment steps: a STAR*D report. Am J Psychiatry.

[4] Goldberg JF, Freeman MP, Balon R, Citrome L, Thase ME, Kane JM (2015). The american society of clinical psychopharmacology survey of psychopharmacologists’ practice patterns for the treatment of mood disorders. Depress Anxiety.

[5] Misdrahi D, Tessier A, Daubigney A, Meissner WG, Schurhoff F, Boyer L (2019). Prevalence of and risk factors for extrapyramidal side effects of antipsychotics: results from the national FACE-SZ cohort. J Clin Psychiatry.

[6] Tu TH, Huang KL, Bai YM, Hsu JW, Su TP, Li CT (2019). Exposure to second-generation antipsychotics and risk of type 2 diabetes mellitus in adolescents and young adults: a nationwide study in Taiwan. J Clin Psychiatry.

[7] Godin O, Leboyer M, Schürhoff F, Llorca PM, Boyer L, Andre M (2018). Metabolic syndrome and illness severity predict relapse at 1-year follow-up in schizophrenia: the FACE-SZ cohort. J Clin Psychiatry.

[8] Bobo WV, Shelton RC (2009). Fluoxetine and olanzapine combination therapy in treatment-resistant major depression: review of efficacy and safety data. Expert Opin Pharmacother.

[9] Silberner J (2019). Ketamine should be available for treatment resistant depression, says FDA panel. BMJ.

[10] Salloum NC, Papakostas GI (2019). Staging treatment intensity and defining resistant depression: historical overview and future directions. J Clin Psychiatry.

[11] Ionescu DF, Papakostas GI (2017). Experimental medication treatment approaches for depression. Transl Psychiatry.

[12] Iovieno N, Papakostas GI (2012). Correlation between different levels of placebo response rate and clinical trial outcome in major depressive disorder: a meta-analysis. J Clin Psychiatry.

[13] Papakostas GI, Østergaard SD, Iovieno N (2015). The nature of placebo response in clinical studies of major depressive disorder. J Clin Psychiatry.

[14] Guidi J, Brakemeier EL, Bockting CLH, Cosci F, Cuijpers P, Jarrett RB (2018). Methodological recommendations for trials of psychological interventions. Psychother Psychosom.

[15] Fava M, Evins AE, Dorer DJ, Schoenfeld DA (2003). The problem of the placebo response in clinical trials for psychiatric disorders: culprits, possible remedies, and a novel study design approach. Psychother Psychosom.

[16] HAMILTON M (1960). A rating scale for depression. J Neurol Neurosurg Psychiatry.

[17] Montgomery SA, Asberg M (1979). A new depression scale designed to be sensitive to change. Br J Psychiatry.

[18] Papakostas GI, Fava M (2009). Does the probability of receiving placebo influence clinical trial outcome? A meta-regression of double-blind, randomized clinical trials in MDD. Eur Neuropsychopharmacol.

[19] Iovieno N, Papakostas GI (2012). Does the presence of an open-label antidepressant treatment period influence study outcome in clinical trials examining augmentation/combination strategies in treatment partial responders/nonresponders with major depressive disorder?. J Clin Psychiatry.

[20] Papakostas GI, Ionescu DF (2015). Towards new mechanisms: an update on therapeutics for treatment-resistant major depressive disorder. Mol Psychiatry.

[21] Papakostas GI, Ionescu DF (2014). Updates and trends in the treatment of major depressive disorder. J Clin Psychiatry.

