# Supplementary material for: Success and efficiency of phase 2/3 adjunctive trials for MDD funded by industry: a systematic review
Source: Mol Psychiatry. 2020 Jan 27;25(9):1967–74. doi: 10.1038/s41380-020-0646-3 (PMC7473846; doi:10.1038/s41380-020-0646-3)
Supplement: Supplementary file 1 — Supplemental Table 1 [file 41380_2020_646_MOESM1_ESM.pdf]

Supplemental Table 1: Studies Characteristics

Study Number	Study	Lead-in?	Type of lead-in (OL, SB, DB)	Duration of lead-in (weeks)	Total N enrolled in Lead-in	Duration to primary endpoint (weeks)	Agent	Dose (mg)	Scale	N Drug	N Control	Positive Primary Outcome?
1[1]	Papakostas GI et al, 2012-A (SPCD stage I)	N			148	4	l-methylfolate	7.5	HAMD	33	98	
1	Papakostas GI et al, 2012-A (SPCD stage II)	Y	DB (SPCD)	4		4	l-methylfolate	7.5	HAMD	30	30	N
2[1]	Papakostas GI et al, 2012-B (SPCD stage I)	N			75	4	l-methylfolate	15	HAMD	19	56	
2	Papakostas GI et al, 2012-B (SPCD stage II)	Y	DB (SPCD)	4		4	l-methylfolate	15	HAMD	19	56	Y
3[2]	Fava M et al, 2012 (SPCD stage I)	N			221	4	aripiprazole	2	MADRS	54	167	
3	Fava M et al, 2012 (SPCD stage II)	Y	DB (SPCD)	4		4	aripiprazole	2	MADRS	61	63	N
4[3]	Fava M et al, 2016 (SPCD stage I)	N			142	4	alks-5461	2/2	MADRS	24	98	
4	Fava M et al, 2016 (SPCD stage II)	Y	DB (SPCD)	4		4	alks-5461	2/2	MADRS	23	20	Y
4	Fava M et al, 2016 (SPCD stage I)	N				4	alks-5461	8/8	MADRS	19	98	
4	Fava M et al, 2016 (SPCD stage II)	Y	DB (SPCD)	4		4	alks-5461	8/8	MADRS	22	20	N
5[4]	Fava M et al, 2018-A (SPCD stage I)	N			385	6	alks-5461	0.5/0.5	MADRS	59	266	
5	Fava M et al, 2018-A (SPCD stage II)	Y	DB (SPCD)	5		6	alks-5461	0.5/0.5	MADRS	56	56	N
5	Fava M et al, 2018-A (SPCD stage I)	N				6	alks-5461	2/2	MADRS	60	266	
5	Fava M et al, 2018-A (SPCD stage II)	Y	DB (SPCD)	5		6	alks-5461	2/2	MADRS	56	56	N
6[4]	Fava M et al, 2018-B (SPCD stage I)	N			407	6	alks-5461	1/1	MADRS	63	281	
6	Fava M et al, 2018-B (SPCD stage II)	Y	DB (SPCD)	5		6	alks-5461	1/1	MADRS	62	62	N
6	Fava M et al, 2018-B (SPCD stage I)	N				6	alks-5461	2/2	MADRS	63	281	
6	Fava M et al, 2018-B (SPCD stage II)	Y	DB (SPCD)	5		6	alks-5461	2/2	MADRS	63	62	Y
7[5]	Fava M et al, 2019 (SPCD stage I)	N			207	5	Pimavanserin	34	HAMD	52	155	
7	Fava M et al, 2019 (SPCD stage II)	Y	DB (SPCD)	5		5	Pimavanserin	34	HAMD	29	29	Y
8[6]	Baeur M et al, 2009-A	N	None	None	None	6	Quetiapine ER	150	MADRS	166	160	Y
8	Baeur M et al, 2009-B	N	None	None	None	6	Quetiapine ER	300	MADRS	161	160	Y
9[7]	El Khalili N, 2010-A	N	None	None	None	6	Quetiapine ER	150	MADRS	143	143	N
9	El Khalili N, 2010-B	N	None	None	None	6	Quetiapine ER	300	MADRS	146	143	Y
10[8]	Durgam S, 2016-A	N	None	None	None	8	Cariprazine	1 to 2	MADRS	273	264	N
10	Durgam S, 2016-B	N	None	None	None	8	Cariprazine	2 to 4.5	MADRS	271	264	Y
11[9]	Connor KM, 2017	N	None	None	None	6	Filorexant	10	MADRS	64	64	N
12[10]	Quiroz JA, 2016-A	N	None	None	None	6	Basimglurant MR	0.5	MADRS	112	109	N
12	Quiroz JA, 2016-B	N	None	None	None	6	Basimglurant MR	1.5	MADRS	111	109	N
13[11]	Umbricht, 2015-A	N	None	None	None	6	RG1578	5	MADRS	89	86	N
13	Umbricht, 2015-B	N	None	None	None	6	RG1578	15	MADRS	88	86	N
13	Umbricht, 2015-C	N	None	None	None	6	RG1578	30	MADRS	47	86	N
14[12]	Fava M, 2018-A	Y	OL	8	502	8	Cariprazine	0.1 to 0.3	MADRS	76	81	N
14	Fava M, 2018-B	Y	OL	8		8	Cariprazine	1 to 2	MADRS	73	81	N
15[13]	Mahmoud RA, 2007	Y	OL	4	463	6	Risperidone	1 to 2	HAMD	137	131	Y
16[14]	Fava M, 2015	Y	OL	8	297	6	CP-601,927	1 to 4	MADRS	62	63	N
17[15]	Vieta E, 2014(1)	Y	OL	8	710	8	Dexmecamylamine	2 to 8	MADRS	151	157	N
18[15]	Vieta E, 2014(2)	Y	OL	8	617	8	Dexmecamylamine	2 to 8	MADRS	145	145	N
19[16]	Barbee JG, 2011	Y	OL	Not mentioned	180	10	Lamotrigine	up to 400	MADRS	48	48	N
20[17]	Thase ME, 2007(1)	Y	OL	8	1313	8	Olanazapine/Fluoxetine	6 to 18/50	MADRS	N/A	N/A	N
21[17]	Thase ME, 2007(2)	Y	OL	8		8	Olanazapine/Fluoxetine	6 to 18/50	MADRS	N/A	N/A	Y
22[18]	Moeller, 2015(1)-A	Y	OL	8	1289	8	Dexmecamylamine	0.4	MADRS	173	174	N
22	Moeller, 2015(1)-B	Y	OL	8		8	Dexmecamylamine	2	MADRS	174	174	N
22	Moeller, 2015(1)-C	Y	OL	8		8	Dexmecamylamine	4	MADRS	170	174	N
23[18]	Moeller, 2015(2)-A	Y	OL	8	1317	8	Dexmecamylamine	0.1	MADRS	155	157	N
23	Moeller, 2015(2)-B	Y	OL	8		8	Dexmecamylamine	1	MADRS	156	157	N
23	Moeller, 2015(2)-C	Y	OL	8		8	Dexmecamylamine	4	MADRS	152	157	N
24[19]	Berman M., 2007	Y	SB	8	781	6	Aripiprazole	2 to 15	MADRS	181	172	Y
25[20]	Berman M., 2009	Y	SB	8	827	6	Aripiprazole	2 to 20	MADRS	174	169	Y
26[21]	Kamijima K, 2013-A	Y	SB	8	1115	6	Aripiprazole	3	MADRS	197	195	Y
26	Kamijima K, 2013-B	Y	SB	8		6	Aripiprazole	3 to 15	MADRS	194	195	Y
27[22]	Marcus RN, 2008	Y	SB	8	830	6	Aripiprazole	2 to 20	MADRS	185	184	Y
28[23]	Earley WR, 2018	Y	SB	8	1022	8	Cariprazine	1.5 to 4.5	MADRS	267	258	N
29[24]	Hobart M, 2018	Y	SB	8	837	6	Brexpiprazole	2	MADRS	191	202	Y
30[25]	Thase ME, 2019(1)	Y	SB	8		6	Brexpiprazole	0.15	MADRS	45	89	N
30	Thase ME, 2019(1)	Y	SB	8		6	Brexpiprazole	0.25 to 0.75	MADRS	94	89	N
30	Thase ME, 2019(1)	Y	SB	8	850	6	Brexpiprazole	1 to 2	MADRS	90	89	Y
31[25]	Thase ME, 2019(2)	Y	SB	8	773	6	Brexpiprazole	1 to 3	MADRS	158	147	N
32[26]	Thase ME, 2015-A	Y	SB	8	1532	6	Brexpiprazole	1	MADRS	211	203	N
32	Thase ME, 2015-B	Y	SB	8		6	Brexpiprazole	3	MADRS	213	203	Y
33[27]	Thase ME, 2015 (2)	Y	SB	8	826	6	Brexpiprazole	2	MADRS	175	178	Y
34[28]	Richards C, 2016(1)	Y	SB	8	1239	8	Lisdexamfetamine	20 to 70	MADRS	200	209	N
35[28]	Richards C, 2016 (2)	Y	SB	8	1095	8	Lisdexamfetamine	20 to 70	MADRS	200	213	N
36[29]	Richards C, 2017-A	Y	SB	8	1178	8	Lisdexamfetamine	10	MADRS	77	78	N
36	Richards C, 2017-B	Y	SB	9		8	Lisdexamfetamine	30	MADRS	76	78	N
36	Richards C, 2017-C	Y	SB	10		8	Lisdexamfetamine	50	MADRS	78	78	N
36	Richards C, 2017-D	Y	SB	11		8	Lisdexamfetamine	70	MADRS	80	78	N
37[30]	Trivedi MH, 2013	Y	SB	8	239	6	Lisdexamfetamine	20 to 50	MADRS	65	64	Y
38[31]	Kamijima 2018	Y	SB	8	899	6	Aripiprazole/Sertraline	3 to 12/100	MADRS	208	203	Y
39[32]	Hobart M, 2018 (2)-A	Y	DB	8 to 10	2174	6	Brexpiprazole	2 to 3	MADRS	191	205	Y
39	Hobart M, 2018 (2)-B	Y	DB	8 to 10		6	Quetiapine XR	150 to 300	MADRS	99	205	N
40[33]	Ball S, 2014	Y	DB	2	227	8	Edivoxetine	6 to 18	MADRS	63	68	N
41[34]	Ball S, 2016-A	Y	DB	3	1416	8	Edivoxetine	12	MADRS	230	240	N
41	Ball S, 2016-B	Y	DB	3		8	Edivoxetine	18	MADRS	230	240	N
42[34]	Ball S, 2016-A	Y	DB	3	1480	8	Edivoxetine	6	MADRS	220	228	N
42	Ball S, 2016-B	Y	DB	3		8	Edivoxetine	12 to 18	MADRS	231	228	N
43[34]	Ball S, 2016	Y	DB	4	1056	8	Edivoxetine	12 to 18	MADRS	225	218	N
44[35]	Targum SD, 2018	Y	DB	2	376	6	SAMe	800	MADRS	118	116	N
45[36]	FORWARD 3	Y	DB	4	429	6	ALKS-5461	2/2	MADRS	149	148	N
46[37]	Bauer, 2019	Y	DB	8	1986	24	Brexpiprazole	1 to 3	MADRS	444	442	N

N=No; Y=Yes; OL=Open-label; SB=Single-blind; DB=Double-blind

Studies References

1. Papakostas GI, Shelton RC, Zajecka JM, et al. L-methylfolate as adjunctive therapy for SSRI-resistant major depression: results of two randomized, double-blind, parallel-sequential trials. *Am J Psychiatry.* 2012;169(12):1267-1274.
2. Fava M, Mischoulon D, Iosifescu D, et al. A double-blind, placebo-controlled study of aripiprazole adjunctive to antidepressant therapy among depressed outpatients with inadequate response to prior antidepressant therapy (ADAPT-A Study). *Psychother Psychosom.*

2012;81(2):87-97.
3. Fava M, Memisoglu A, Thase ME, et al. Opioid Modulation With Buprenorphine/Samidorphan as Adjunctive Treatment for Inadequate Response to Antidepressants: A Randomized Double-Blind Placebo-Controlled Trial. *Am J Psychiatry.* 2016;173(5):499-508.
4. Fava M, Thase ME, Trivedi MH, et al. Opioid system modulation with buprenorphine/samidorphan combination for major depressive disorder: two randomized controlled studies. *Mol Psychiatry.* 2018.
5. Fava M, Dirks B, Freeman MP, et al. A Phase 2, Randomized, Double-Blind, Placebo-Controlled Study of Adjunctive Pimavanserin in Patients With Major Depressive Disorder and an Inadequate Response to Therapy (CLARITY). *J Clin Psychiatry.* 2019;80(6).
6. Bauer M, Pretorius HW, Constant EL, Earley WR, Szamosi J, Brecher M. Extended-release quetiapine as adjunct to an antidepressant in patients with major depressive disorder: results of a randomized, placebo-controlled, double-blind study. *J Clin Psychiatry.* 2009;70(4):540-549.
7. El-Khalili N, Joyce M, Atkinson S, et al. Extended-release quetiapine fumarate (quetiapine XR) as adjunctive therapy in major depressive disorder (MDD) in patients with an inadequate response to ongoing antidepressant treatment: a multicentre, randomized, double-blind, placebo-controlled study. *Int J Neuropsychopharmacol.* 2010;13(7):917-932.
8. Durgam S, Earley W, Guo H, et al. Efficacy and safety of adjunctive cariprazine in inadequate responders to antidepressants: a randomized, double-blind, placebo-controlled study in adult patients with major depressive disorder. *J Clin Psychiatry.* 2016;77(3):371-378.
9. Connor KM, Ceesay P, Hutzelmann J, et al. Phase II Proof-of-Concept Trial of the Orexin Receptor Antagonist Filorexant (MK-6096) in Patients with Major Depressive Disorder. *Int J Neuropsychopharmacol.* 2017;20(8):613-618.
10. Quiroz JA, Tamburri P, Deptula D, et al. Efficacy and Safety of Basimglurant as Adjunctive Therapy for Major Depression: A Randomized Clinical Trial. *JAMA Psychiatry.* 2016;73(7):675-684.
11. Umbricht D., Niggli M., Sanwald-Ducray P., Deptula D. P.2.f.021 Results of a double-blind placebo-controlled study of the antidepressant effects of the mGLU2 negative allosteric modulator RG1578. *European Neuropsychopharmacology.* 2015; 25(2):S447. In.
12. Fava M, Durgam S, Earley W, et al. Efficacy of adjunctive low-dose cariprazine in major depressive disorder: a randomized, double-blind, placebo-controlled trial. *Int Clin Psychopharmacol.* 2018;33(6):312-321.
13. Mahmoud RA, Pandina GJ, Turkoz I, et al. Risperidone for treatment-refractory major depressive disorder: a randomized trial. *Ann Intern Med.* 2007;147(9):593-602.
14. Fava M, Ramey T, Pickering E, Kinrys G, Boyer S, Altstiel L. A randomized, double-blind, placebo-controlled phase 2 study of the augmentation of a nicotinic acetylcholine receptor partial agonist in depression: is there a relationship to leptin levels? *J Clin Psychopharmacol.* 2015;35(1):51-56.
15. Vieta E, Thase ME, Naber D, et al. Efficacy and tolerability of flexibly-dosed adjunct TC-5214 (dexmecamylamine) in patients with major depressive disorder and inadequate response to prior antidepressant. *Eur Neuropsychopharmacol.* 2014;24(4):564-574.
16. Barbee JG, Thompson TR, Jamhour NJ, et al. A double-blind placebo-controlled trial of lamotrigine as an antidepressant augmentation agent in treatment-refractory unipolar depression. *J Clin Psychiatry.* 2011;72(10):1405-1412.
17. Thase ME, Corya SA, Osuntokun O, et al. A randomized, double-blind comparison of olanzapine/fluoxetine combination, olanzapine, and fluoxetine in treatment-resistant major depressive disorder. *J Clin Psychiatry.* 2007;68(2):224-236.
18. Möller HJ, Demyttenaere K, Olausson B, et al. Two Phase III randomised double-blind studies of fixed-dose TC-5214 (dexmecamylamine) adjunct to ongoing antidepressant therapy in patients with major depressive disorder and an inadequate response to prior antidepressant therapy. *World J Biol Psychiatry.* 2015;16(7):483-501.
19. Berman RM, Marcus RN, Swanink R, et al. The efficacy and safety of aripiprazole as adjunctive therapy in major depressive disorder: a multicenter, randomized, double-blind, placebo-controlled study. *J Clin Psychiatry.* 2007;68(6):843-853.
20. Berman RM, Fava M, Thase ME, et al. Aripiprazole augmentation in major depressive disorder: a double-blind, placebo-controlled study in patients with inadequate response to antidepressants. *CNS Spectr.* 2009;14(4):197-206.
21. Kamijima K, Higuchi T, Ishigooka J, et al. Aripiprazole augmentation to antidepressant therapy in Japanese patients with major depressive disorder: a randomized, double-blind, placebo-controlled study (ADMIRE study). *J Affect Disord.* 2013;151(3):899-905.
22. Marcus RN, McQuade RD, Carson WH, et al. The efficacy and safety of aripiprazole as adjunctive therapy in major depressive disorder: a second multicenter, randomized, double-blind, placebo-controlled study. *J Clin Psychopharmacol.* 2008;28(2):156-165.
23. Earley WR, Guo H, Németh G, Harsányi J, Thase ME. Cariprazine Augmentation to Antidepressant Therapy in Major Depressive Disorder: Results of a Randomized, Double-Blind, Placebo-Controlled Trial. *Psychopharmacol Bull.* 2018;48(4):62-80.
24. Hobart M, Skuban A, Zhang P, et al. A Randomized, Placebo-Controlled Study of the Efficacy and Safety of Fixed-Dose Brexpiprazole 2 mg/d as Adjunctive Treatment of Adults With Major Depressive Disorder. *J Clin Psychiatry.* 2018;79(4).
25. Thase ME, Zhang P, Weiss C, Meehan SR, Hobart M. Efficacy and safety of brexpiprazole as adjunctive treatment in major depressive disorder: overview of four short-term studies. *Expert Opin Pharmacother.* 2019;20(15):1907-1916.
26. Thase ME, Youakim JM, Skuban A, et al. Adjunctive brexpiprazole 1 and 3 mg for patients with major depressive disorder following inadequate response to antidepressants: a phase 3, randomized, double-blind study. *J Clin Psychiatry.* 2015;76(9):1232-1240.
27. Thase ME, Youakim JM, Skuban A, et al. Efficacy and safety of adjunctive brexpiprazole 2 mg in major depressive disorder: a phase 3, randomized, placebo-controlled study in patients with inadequate response to antidepressants. *J Clin Psychiatry.* 2015;76(9):1224-1231.
28. Richards C, McIntyre RS, Weisler R, et al. Lisdexamfetamine dimesylate augmentation for adults with major depressive disorder and inadequate response to antidepressant monotherapy: Results from 2 phase 3, multicenter, randomized, double-blind, placebo-controlled studies. *J Affect Disord.* 2016;206:151-160.
29. Richards C, Iosifescu DV, Mago R, et al. A randomized, double-blind, placebo-controlled, dose-ranging study of lisdexamfetamine dimesylate augmentation for major depressive disorder in adults with inadequate response to antidepressant therapy. *J Psychopharmacol.* 2017;31(9):1190-1203.
30. Trivedi MH, Cutler AJ, Richards C, et al. A randomized controlled trial of the efficacy and safety of lisdexamfetamine dimesylate as augmentation therapy in adults with residual symptoms of major depressive disorder after treatment with escitalopram. *J Clin Psychiatry.* 2013;74(8):802-809.
31. Kamijima K, Kimura M, Kuwahara K, Kitayama Y, Tadori Y. Randomized, double-blind comparison of aripiprazole/sertraline combination and placebo/sertraline combination in patients with major depressive disorder. *Psychiatry Clin Neurosci.* 2018;72(8):591-601.
32. Hobart M, Skuban A, Zhang P, et al. Efficacy and safety of flexibly dosed brexpiprazole for the adjunctive treatment of major depressive disorder: a randomized, active-referenced, placebo-controlled study. *Curr Med Res Opin.* 2018;34(4):633-642.
33. Ball S, Dellva MA, D'Souza DN, Marangell LB, Russell JM, Goldberger C. A double-blind, placebo-controlled study of edivoxetine as an adjunctive treatment for patients with major depressive disorder who are partial responders to selective serotonin reuptake inhibitor treatment. *J Affect Disord.* 2014;167:215-223.
34. Ball SG, Ferguson MB, Martinez JM, et al. Efficacy outcomes from 3 clinical trials of edivoxetine as adjunctive treatment for patients with major depressive disorder who are partial responders to selective serotonin reuptake inhibitor treatment. *J Clin Psychiatry.* 2016;77(5):635-642.
35. Targum SD, Cameron BR, Ferreira L, MacDonald ID. An augmentation study of MSI-195 (S-adenosylmethionine) in Major Depressive Disorder. *J Psychiatr Res.* 2018;107:86-96.
36. Alkermes Inc. A Study of ALKS 5461 for the Treatment of Major Depressive Disorder (MDD) - the FORWARD-3 Study. Clinicaltrials.gov Identifier NCT02158546. In.
37. Bauer M, Hefting N, Lindsten A, Josiassen MK, Hobart M. A randomised, placebo-controlled 24-week study evaluating adjunctive brexpiprazole in patients with major depressive disorder. *Acta Neuropsychiatr.* 2019;31(1):27-35.
