# Supplementary material for: Success and efficiency of phase 2/3 adjunctive trials for MDD funded by industry: a systematic review
Source: Mol Psychiatry. 2020 Jan 27;25(9):1967–74. doi: 10.1038/s41380-020-0646-3 (PMC7473846; doi:10.1038/s41380-020-0646-3)
Supplement: Supplementary file 2 — Supplemental Figure 1 [file 41380_2020_646_MOESM2_ESM.pptx]

## Slide 1
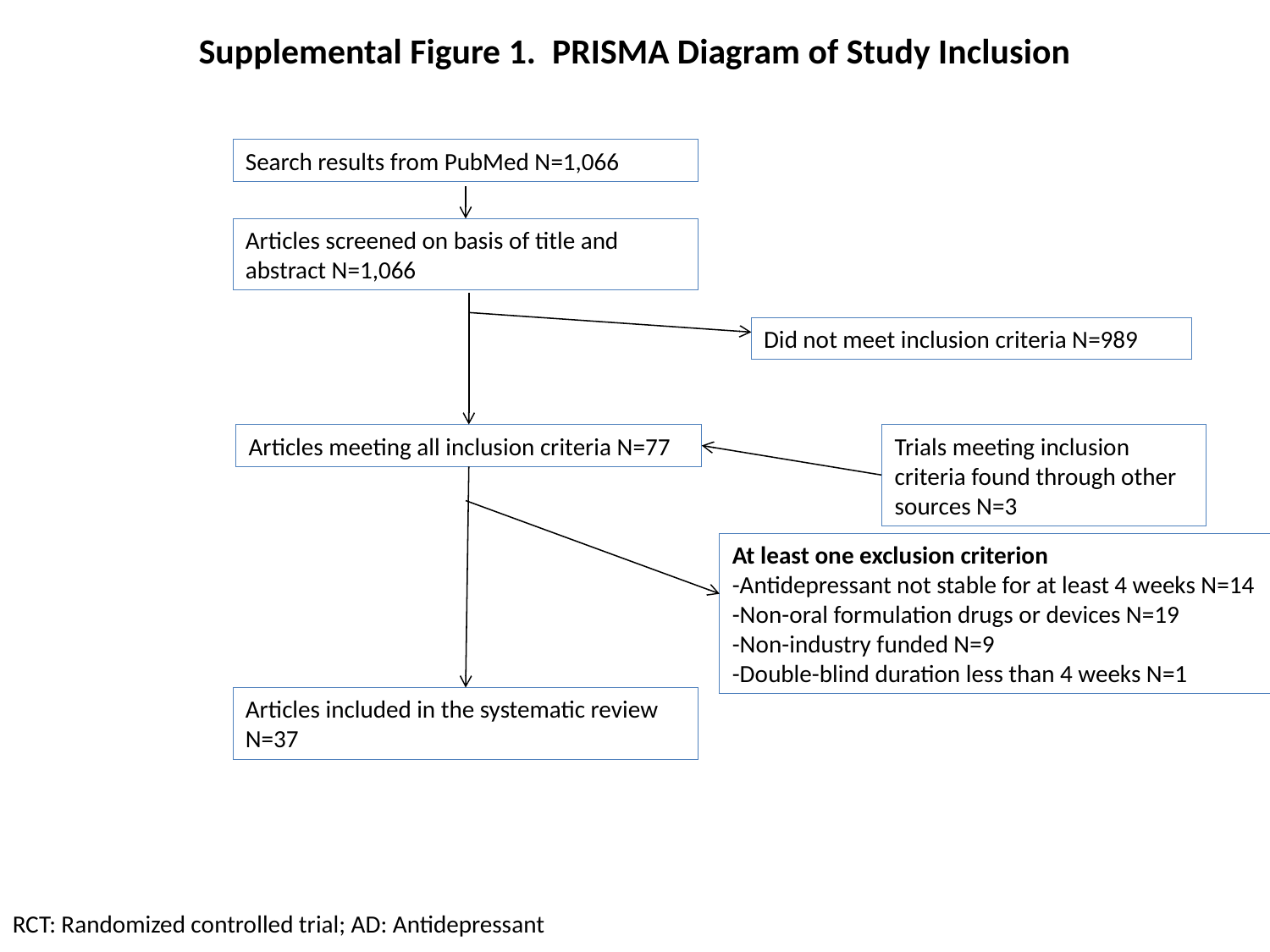

Supplemental Figure 1. PRISMA Diagram of Study Inclusion
Search results from PubMed N=1,066
Articles screened on basis of title and abstract N=1,066
Did not meet inclusion criteria N=989
Articles meeting all inclusion criteria N=77
Trials meeting inclusion criteria found through other sources N=3
At least one exclusion criterion
-Antidepressant not stable for at least 4 weeks N=14
-Non-oral formulation drugs or devices N=19
-Non-industry funded N=9
-Double-blind duration less than 4 weeks N=1
Articles included in the systematic review N=37
RCT: Randomized controlled trial; AD: Antidepressant
