# Supplementary material for: Success and efficiency of phase 2/3 adjunctive trials for MDD funded by industry: a systematic review
Source: Mol Psychiatry. 2020 Jan 27;25(9):1967–74. doi: 10.1038/s41380-020-0646-3 (PMC7473846; doi:10.1038/s41380-020-0646-3)
Supplement: Supplementary file 3 — Supplemental Figure 2 [file 41380_2020_646_MOESM3_ESM.pptx]

## Slide 1
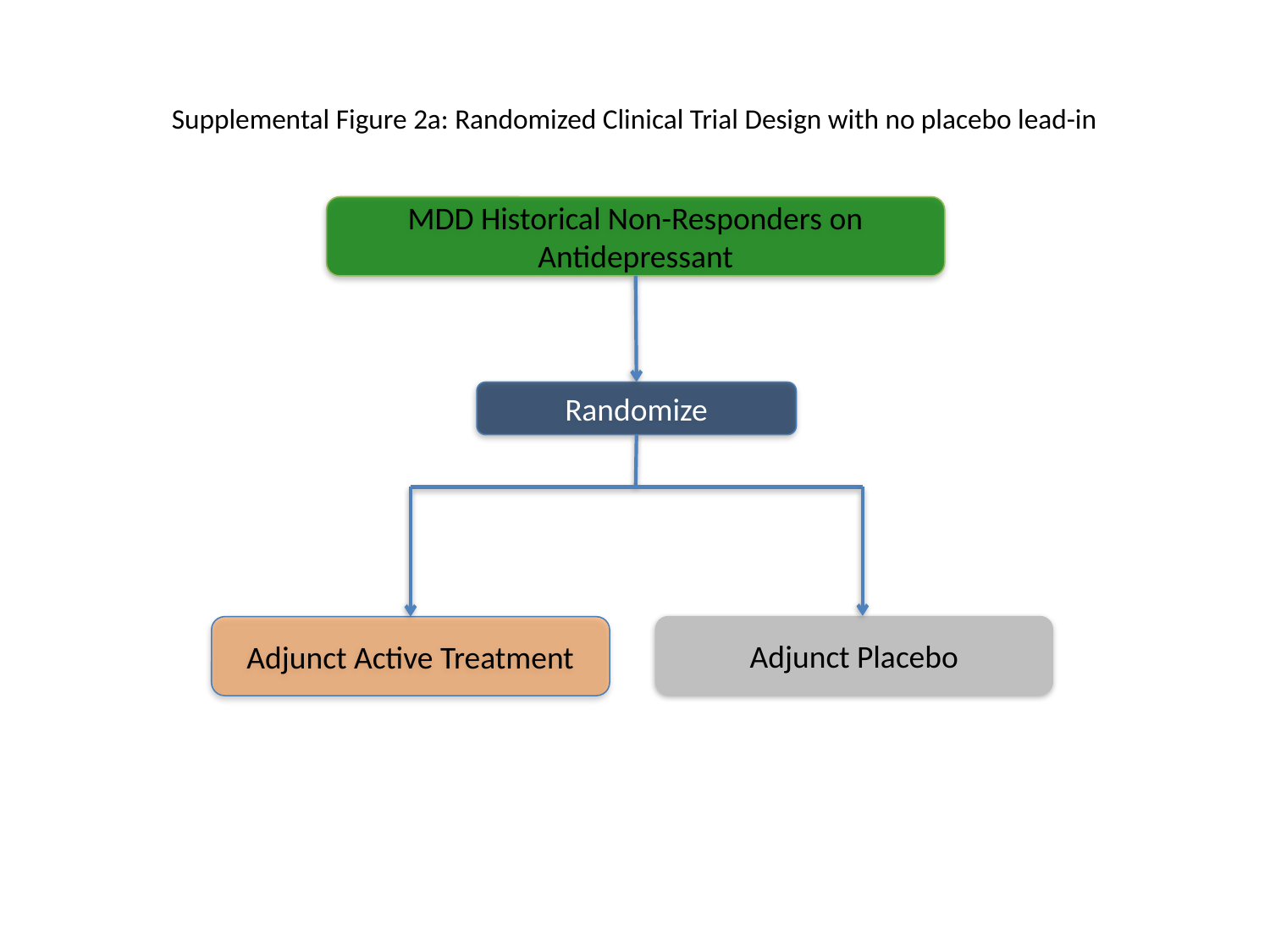

# Supplemental Figure 2a: Randomized Clinical Trial Design with no placebo lead-in
MDD Historical Non-Responders on Antidepressant
Randomize
Adjunct Placebo
Adjunct Active Treatment

## Slide 2
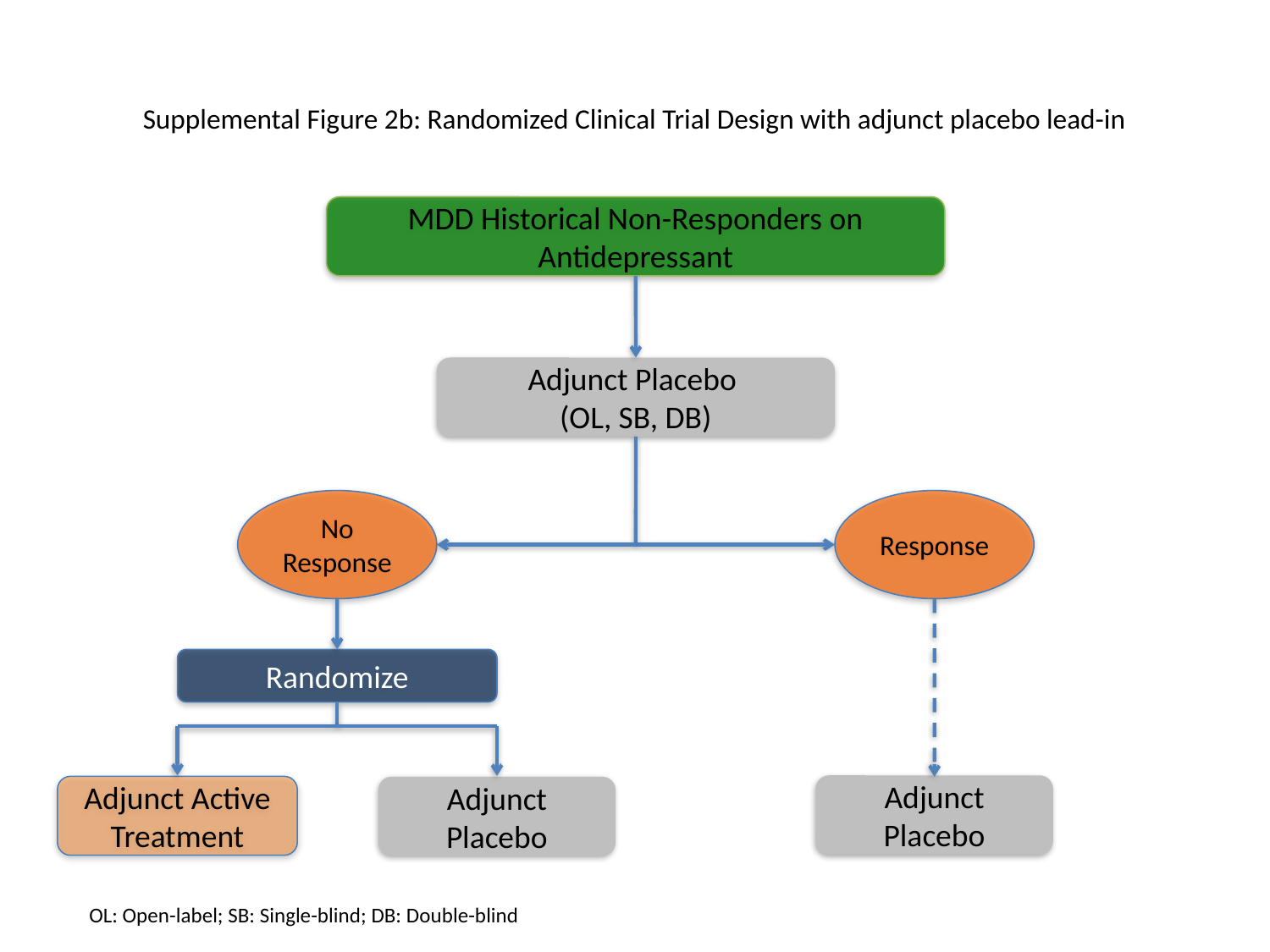

# Supplemental Figure 2b: Randomized Clinical Trial Design with adjunct placebo lead-in
MDD Historical Non-Responders on Antidepressant
Adjunct Placebo
(OL, SB, DB)
No Response
Response
Randomize
Adjunct Placebo
Adjunct Active Treatment
Adjunct Placebo
OL: Open-label; SB: Single-blind; DB: Double-blind

## Slide 3
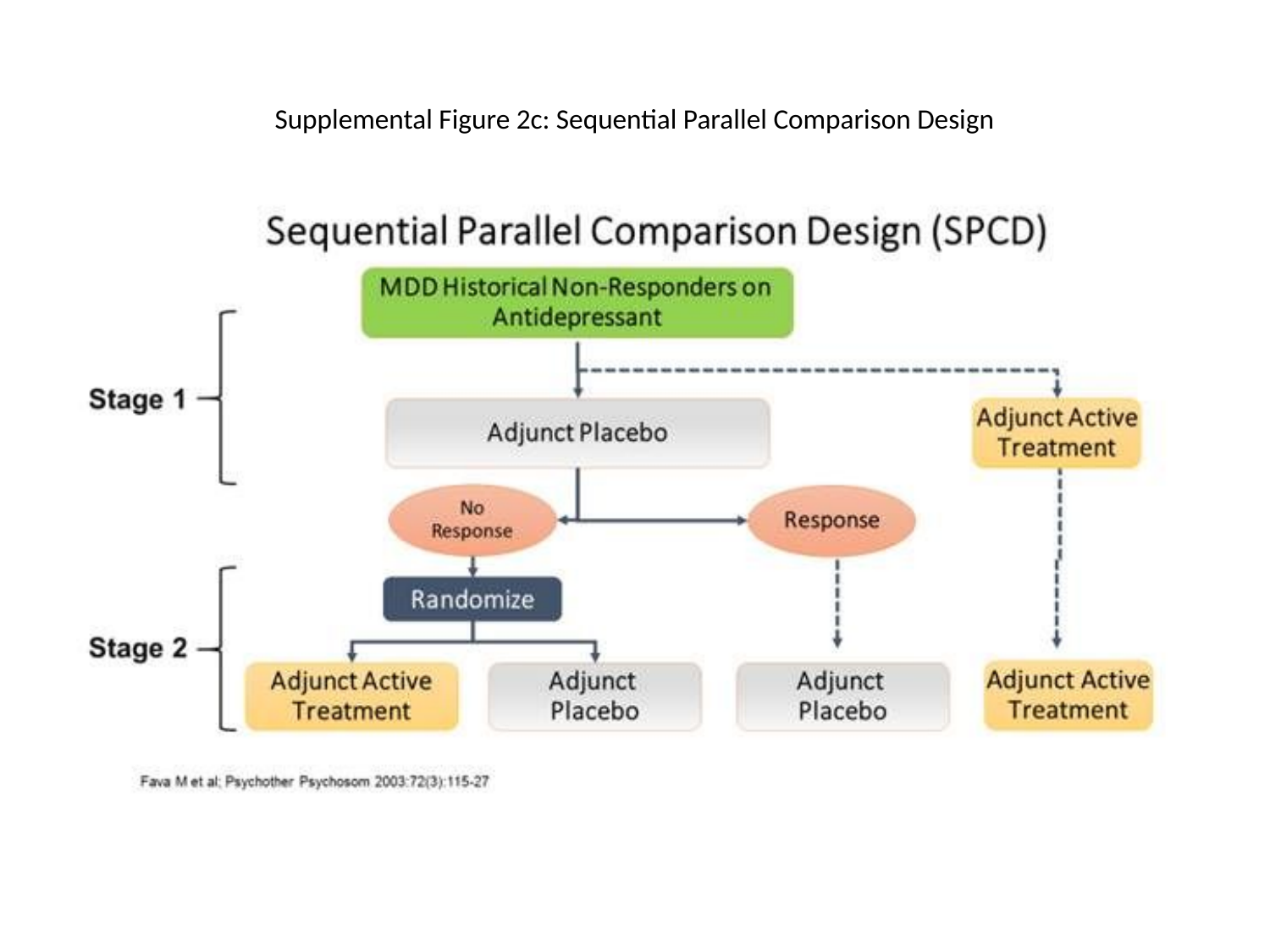

# Supplemental Figure 2c: Sequential Parallel Comparison Design
